# Association between second-hand smoke and psychological well-being amongst non-smoking wageworkers in Republic of Korea

**DOI:** 10.1186/s40557-016-0130-3

**Published:** 2016-09-20

**Authors:** Seong-Jin Kim, Dirga Kumar Lamichhane, Shin-Goo Park, Bum-Joon Lee, So-Hyun Moon, Sung-Min Park, Hyun-Suk Jang, Hwan-Cheol Kim

**Affiliations:** 1 Department of Occupational and Environmental Medicine, Inha University Hospital, Incheon, Republic of Korea; 2 Department of Social and Preventive Medicine, School of Medicine, Inha University, Incheon, Republic of Korea; 3 Department of Occupational and Environmental Medicine, School of Medicine, Inha University, Incheon, Republic of Korea

**Keywords:** Psychological well-being, Second-hand Smoke, Wageworkers, WHO-5 index

## Abstract

**Background:**

Worldwide, exposure to second-hand smoke (SHS) has been responsible for more than 0.6 million deaths and 10.9 million disability-adjusted life years (DALYS) lost in never smokers in 2004. The world health organization (WHO) reported smoking-related death of 58,000 per year in South Korea. There is recent emerging evidence of the associations of SHS exposure with anxiety or depression and poor mental health. Although exposure to second-hand smoke (SHS) has been associated with various physical health conditions and mental health, we are unaware of any studies examining its association with psychological well-being as mental factor. This study aimed to investigate the association between self-reported exposure to SHS and well-being among non-smoking wageworkers.

**Methods:**

The Third Korean Working Conditions Survey (KWCS, 2011) was conducted on a representative sample of economically active population aged 15 years or over, who were either employees or self-employed at the time of interview. In this study, after removing inconsistent data, 19,879 non-smoking wageworkers among 60,054 workers were participated. Psychological well-being was measured through the WHO-Five Well-Being Index (1998 version). Univariate and multiple logistic regression models were used to examine the association of SHS exposure with psychological well-being.

**Results:**

The unadjusted OR of poor psychological well-being (OR: 1.594, 95 % CI: 1.421-1.787) was significantly higher for SHS exposure group compared to non-exposure group. Multiple logistic regression analysis results indicated that these relationships were still significant after adjusting for potential confounders (adjusted OR: 1.330, 95 % CI: 1.178-1.502).

**Conclusions:**

Exposure to SHS was associated with poor well-being measured by the WHO-5 well-being index, indicating the importance of reducing SHS exposure at the workplace for psychological well-being amongst non-smoking wageworkers.

## Background

Worldwide, exposure to second-hand smoke (SHS) has been responsible for more than 0.6 million deaths and 10.9 million disability-adjusted life years (DALYS) lost in never smokers in 2004 [1]. The world health organization (WHO) reported smoking-related death of 58,000 per year in South Korea [2]. Epidemiological studies have demonstrated that SHS is associated with numerous physical health consequences, including cardiovascular disease, stroke, lung cancer, chronic respiratory symptoms and impaired pulmonary function [3–7]. There is recent emerging evidence of the associations of SHS exposure with anxiety or depression and poor mental health [8–10].

Although workplace smoking ban legislation has been enforced in Korea beginning in 2003, it still has a considerable smoking rate of 42.3 % reported among men and 5.6 % among women [11]. In addition, SHS exposure is common at the workplace; in 2013, 57.2 % of adult men and 38.7 % of adult women who never smoked had exposed to SHS at the workplace in Korea [12]. As it is shown that SHS can lead to impairment of health-related qualities of life and depression, vulnerabilities due to low well-being and potential mental health problem among never smoker exposed to SHS are of concern [13, 14].

The 5-item WHO well-being index (WHO-5), a short and generic global rating scale measuring subjective well-being, is considered as an appropriate measurement of a subject’s physical, mental, and social health because it encompasses the absence of both illness and negative emotions [15]. Well-being is so closely related to mental health that subjects with a low WHO-Five well-being index score are often recommended for depression screening tests [16]. Well-being has been linked with physical health by several studies, demonstrating that satisfied individuals have stronger immune systems and enjoy better physical health [17–19]. Some studies have shown the association between health- related quality of life and SHS exposure among never smokers [13, 14, 20]. There has been few studies that examined the association between SHS exposure and mental health and are mostly focused on the relation between SHS exposure and psychological distress in adult never smokers. The relationship between SHS exposure and mental function is biologically plausible because nicotine is known to affect psychophysiological pathways that are relevant to mental health such as the dopaminergic system, adrenocortical function, and activation of neuroimmunological pathways that have been linked to depression [9, 21–24]. Nicotine amplifies dopamine release during phasic activity in the striatum. Consequently, nicotine imposes genetic effects on phasic dopamine regulation which are likely to affect depression or anxiety. Seccareccia et al. found that serum cotinine as a marker of SHS exposure in epidemiological studies. The results show serum cotinine of non-smokers (SHS exposed: 4.4 ng/ml, SHS non-exposed: 2.8 ng/ml) and smokers (277.3 ng/ml) [25]. In other study, considering detection limit of serum cotinine, there is a study that show the association between SHS exposure and depression by checking serum cotinine of smokers and non-smokers. Although serum cotinine values above the cut-off of 1 ng/ml were not related to depressive problem, they tried to show the association between cotinine levels and depressive problem for SHS exposure [26]. Some studies have shown that mental disorders and cognitive impairment in non-smoking children and adolescents [10, 27]. However, there is a lack of research on the influence of SHS on psychological well-being as measured by the WHO-5-item wellbeing index.

This study aimed to investigate the association between self-reported exposure to SHS and well-being among non-smoking wageworkers, using a nationally representative Korean sample from the third wave of the Korean Working Conditions Survey.

## Methods

### Study population

This study was based on the Third Korean Working Conditions Survey [27]. The purpose of the survey was to gather comprehensive information on Korean working conditions to shed light the nature and types of changes affecting the workforce and the quality of work-life for employees. The survey was conducted in 2011 on a representative sample of economically active population aged 15 years or over, who were either wageworkers or self-employed at the time of interview. Those who were retired and unemployed, as well as housewives and students, were excluded in the survey. In this study, after removing inconsistent data, 19,879 non-smoking wageworkers among 60,054 workers were included. The Institutional Review Board of Inha University Hospital approved the study protocol.

### SHS exposure

Exposure to SHS at the workplace was assessed by the following questions: Are you currently exposed to cigarette smoke by other people at work? Respondents answered according to a seven-point scale that included the following answer options: all of the working time, almost all of the working time, 3/4 of the working time, half of the working time, 1/4 of the working time, almost never, and never. Based on these responses, participants were categorized into exposure to SHS (exposed for ¼ or more of the working hours) and non-exposure (never exposed or almost never exposed) [28].

### WHO-five well-being index

Well-being was evaluated through the WHO-Five Well-Being Index [29]. In 1982, the WHO European Regional Office initiated a European multicenter trial of two different insulin deliverance methods. One of the study objectives was to compare well-being and quality of life in connection with each treatment [30]. Effectiveness of the index has been supported in diagnostic depression screening [16] and evaluation of emotional well-being in patients with chronic diseases including cardiovascular diseases [31] and Parkinson’s disease [32], and in young children [33], and elderly adults [34], as well as diabetic patients.

The index consists of five positively worded items, each of which reflects the presence or absence of well-being and responds to questions about their positive feelings within the last two weeks on 6-point scale (0–5). A raw score lower than 13 out of 30 or an individual item score of 0 or 1 on any of the five items implies a poor well-being. Conversely, those who responded to all the items with a score of 2 or higher and those who had a total score higher than 13 were assigned to the “fair well-being” group [35].

### Potential confounders

We used several other covariates that were likely to be related with well-being globally or in Korea. Previously published reports that showed an association between workplace psychological factors and well-being or variables that could be potential confounders to well-being were also included in the analysis [36, 37]. The following covariates related to socio-economic, structural factors, and health examination were considered: age, education levels, marital status, monthly income, balancing income and expenses, job type, employment status, employment stability, shift work, working hours, smoking area status, physical violence, discrimination, self-rated physical health, hypertension, and obesity. We collapsed self-rated physical health into a dichotomous variable of good (“very good”, “good” or “moderate”) vs. bad (“bad” or “very bad”).

### Statistical analysis

All data were analyzed with the SPSS (ver. 19.0) after encoding was completed. Characteristics of participants with poor and good well-being were compared using Chi-squared test. Univariate and multiple logistic regression models were used to examine the association of SHS exposure with psychological well-being. Adjusted odds ratios were calculated by adjusting for sociodemographic factors, and working condition factors. Furthermore, a multiple logistic regression was stratified by health status (good vs. bad). The bad health status group included subjects that fell into any one of the bad categories in self-rated health, hypertension or obesity. The good health status group included those rated good in self-rated health, no hypertension and no obesity. The level of statistical significance was 0.05.

## Results

### Sociodemographic factors and psychological well-being

The average raw score of the WHO Five Well-being Index in the 19,879 participants was 14.04 (SD: 5.26); 7,200 (36.2 %) were in the poor well-being group, while 12,679 (63.8 %) were in the fair well-being group. 1,532 (7.7 %) were exposed to the smoke from other employees while 18,347 (92.3 %) were not exposed.

The associations between sociodemographic factors and psychological well-being are shown in Table 1. Psychological well-being scores of female had slightly lower than male. Subjects who were over 40 years old had significantly lower psychological well-being. Subjects with lower education levels had significantly higher scores of psychological well-being. Subjects with income lesser than 3 million KRW had significantly lower psychological well-being than those with higher incomes. Subjects who experienced an imbalance between their income and expenses (income-expense balance) had significantly lower psychological well-being than those who faced a balance between income and expenses. The group who were not married had slightly lower scores of psychological well-being than married. The associations between psychological well-being and other mental health factor (health status) are shown. The more good health status had significantly high psychological well-being. The subjects were diagnosed with hypertension had significantly low psychological well-being. The subjects were diagnosed with obesity did not show statistical significance for the association of psychological well-being.

**Table 1:** Sociodemographic factors and Psychological well-being

Variables			Psychological well-being		
		Total	Poor	Fair	
		Number	Number (%)	Number (%)	*p*-value
Total		19,879	7,200 (36.2)	12,679 (63.8)	
Gender	Male	5,963	2,071 (34.7)	3,892 (65.3)	0.004*
	Female	13,916	5,129 (36.9)	8,787 (63.1)	
Age	<29	3,781	1,219 (32.2)	2,562 (67.8)	<0.001*
	30–39	5,705	1,818 (31.9)	3,887 (68.1)	
	40–49	5,710	2,090 (36.6)	3,620 (63.4)	
	50–59	3,115	1,288 (41.3)	1,827 (58.7)	
	>60	1,568	785 (50.1)	783 (49.9)	
Education	Middle school or lower	2,502	1,277 (51.0)	1,225 (49.0)	<0.001*
	High school	7,700	3,019 (39.2)	4,681 (60.8)	
	Junior college	3,620	1,183 (32.7)	2,437 (67.3)	
	College or higher	6,057	1,721 (28.4)	4,336 (71.6)	
Monthly income (KRW)^a^	<1 million	4,032	1,707 (42.3)	2,325 (57.7)	<0.001*
	1–2 million	9,137	3,369 (36.9)	5,768 (63.1)	
	2–3 million	3,913	1,331 (34.0)	2,582 (66.0)	
	>3 million	2,789	790 (28.3)	1,999 (71.7)	
Balancing income and expenses	Difficult	4,175	942 (22.6)	3,233 (77.4)	<0.001*
	Somewhat difficult	5,625	1,753 (31.2)	3,872 (68.8)	
	Somewhat easy	6,914	2,957 (42.8)	3,957 (57.2)	
	Easy	3,165	1,548 (48.9)	1,617 (51.1)	
Marital status	not married	11,362	4,192 (36.9)	7,170 (63.1)	0.023**
	married	8,517	3,008 (35.3)	5,509 (64.7)	
Self-rated physical health	very bad	39	23 (59.0)	16 (41.0)	<0.001*
	bad	605	425 (70.2)	180 (29.8)	
	moderate	5,158	2,620 (50.8)	2,538 (49.2)	
	good	11,957	3,688 (30.8)	8,269 (69.2)	
	very good	2,120	444 (20.9)	1,676 (79.1)	
Hypertension	no	18,906	6,782 (35.9)	12,124 (64.1)	<0.001**
	yes	973	428 (43.0)	555 (57.0)	
Obesity	no	19,520	7,054 (36.1)	12,466 (63.9)	0.085**
	yes	359	146 (40.7)	213 (59.3)	

*Obtained by a chi-squared test

**Obtained by a fisher’s exact test

^a^Obtained data after 8 missing data were excluded

### Working condition factors and psychological well-being

The associations between working condition factors and psychological well-being are shown in Table 2. Blue-collar workers had significantly lower scores of psychological well-being than service workers and white-collar workers. Contingent workers had significantly lower scores of psychological well-being than regular workers. The group with unstable job had significantly lower scores of psychological well-being than the group with stable job. Shift workers had slightly lower scores of psychological well-being than non-shift workers. Subjects who reported that their weekly work hours more than 41 h longer had slightly lower scores of psychological well-being than subjects who worked less than 40 h. Subjects with The absence of smoking area showed significantly lower scores of psychological well-being than the presence of smoking area in or outside working area. The presence of physical violence and discrimination over the past twelve months showed significantly lower scores of psychological well-being than the absence.

**Table 2:** Working condition factors and Psychological well-being

Variables			Psychological well-being		
		Total	Poor	Fair	
		Number	Number (%)	Number (%)	*p*-value
Total		19,879	7,200 (36.2)	12,679 (63.8)	
Job type	blue collar	5,493	2,560 (46.6)	2,933 (53.4)	<0.001*
	service	5,717	2,119 (37.1)	3,598 (62.9)	
	white collar	8,669	2,521 (29.1)	6,148 (70.9)	
Employment type	Contingent	5,736	2,365 (41.2)	3,371 (55.6)	<0.001**
	Regular	14,143	4,835 (34.2)	9,308 (65.8)	
Employment stability^a^	Unstable	1,237	500 (40.4)	737 (59.6)	0.002**
	Stable	18,628	6,692 (35.9)	11,936 (64.1)	
Shift working	no	18,293	6,583 (36.0)	11,710 (64.0)	0.022**
	yes	1,586	617 (38.9)	969 (61.1)	
Weekly working time	<40 h	9,116	3,124 (34.3)	5,992 (65.7)	<0.001*
	41–52 h	6,247	2,307 (36.9)	3,940 (63.1)	
	53–60 h	3,101	1,212 (39.1)	1,889 (60.9)	
	>61 h	1,415	557 (39.4)	858 (60.6)	
Smoking area status^a^	no designated	4,418	1,774 (40.2)	2,644 (59.8)	<0.001*
	in working area	2,371	838 (35.3)	1,533 (64.7)	
	outside working area	9,435	3,160 (33.5)	6,275 (66.5)	
Physical violence	absence	18,892	6,742 (35.7)	12,150 (64.3)	<0.001**
	presence	987	458 (46.4)	529 (53.6)	
Discrimination	absence	17,843	6,383 (35.8)	11,460 (64.2)	<0.001**
	presence	2,036	817 (40.1)	1,219 (59.9)	

*Obtained by a chi-squared test

**Obtained by a fisher’s exact test

^a^Obtained data after missing data were excluded (Employment stability: 14 missing data, Smoking area status: 3655 missing data)

### SHS exposure and psychological well-being

The associations between SHS exposed time and psychological well-being are shown in Table 3. As with the results of the analysis, psychological well-being did not decrease with increasing SHS exposed time. We cannot find a dose-relationship between SHS exposed time and psychological well-being. Further analyses according to a dichotomous variable of non-exposed (almost never and never) vs. exposed (more than ¼ of the working time) revealed that the SHS exposed group was significantly associated with a poor psychological well-being.

**Table 3:** SHS exposure and psychological well-being

			Psychological well-being		
		Total	Poor	Fair	
		Number	Number (%)	Number (%)	*p*-value*
SHS exposure time of working time	No	18,347	6,498 (35.4)	11,849 (64.6)	<0.001
	1/4	955	448 (46.9)	507 (53.1)	
	1/2	312	136 (43.6)	176 (56.4)	
	≥3/4	265	118 (44.5)	147 (55.5)	
SHS exposure	No	18,347	6,498 (35.4)	11,849 (64.6)	<0.001
	Yes	1,532	702 (45.8)	830 (54.2)	

*Obtained by a chi-squared test

Table 4 shows the ORs of poor psychological well-being in relation to exposure to SHS. The unadjusted OR of poor psychological well-being (OR: 1.594, 95 % CI: 1.421-1.787) was significantly higher for SHS exposure group compared to non-exposure group. Multiple logistic regression analysis results indicated that these relationships were still significant after adjusting for potential confounders as described in earlier section (adjusted OR (aOR): 1.330, 95 % CI: 1.178-1.502).

**Table 4:** Odds ratios (OR) and 95 % confidence intervals (CI) for poor psychological well-being by exposure to SHS and stratified by health status

	SHS exposure	Unadjusted		Adjusted^a^	
		OR	95 % CI	OR	95 % CI
All	no	1	-	1	-
	yes	1.594	1.421–1.787	1.330	1.178–1.502
Bad health status^b^	no	1	-	1	-
	yes	1.406	1.004–1.968	1.303	0.907–1.872
Good health status^c^	no	1	-	1	-
	yes	1.576	1.395–1.782	1.335	1.172–1.519

^a^Adjusted for sociodemographic and working condition factors (gender, age, education, monthly income, balancing income and expenses, marital status, job type, employment type, employment stability, shift working, weekly working time, smoking area status, physical violence, discrimination, and health status)

^b^Bad self-rated physical health or hypertension or obesity

^c^Good self-rated physical health, no hypertension, and no obesity

In stratified by health status, the unadjusted ORs of poor psychological well-being (OR: 1.406, 95 % CI: 1.004-1.968 for bad health status and 1.576, 95 % CI: 1.395-1.782 for good health status) were significantly higher for SHS exposure group compared to reference group. Additional adjustment for covariates, did not alter the OR considerably in good health status group (1.335; 95 % CI: 1.172-1.519). Among the bad health status group, however, the OR was not significant (1.303; 95 % CI: 0.907–1.872) (Table 4).

In stratified by age, the unadjusted ORs of poor psychological well-being for <39 (OR: 1.663, 95 % CI: 1.387-1.994), and 40–59 group (OR: 1.472, 95 % CI: 1.250-1.733) were significantly higher for SHS exposure group compared to reference group. Additional adjustment for covariates, did not alter the OR considerably in <39 (OR: 1.398, 95 % CI: 1.152-1.696), and 40–59 group (OR: 1.291, 95 % CI: 1.087-1.535). Among the older than 60, however, the OR was not significant (OR: 1.393, 95 % CI: 0.942-2.059) (Table 5).

**Table 5:** Odds ratios (OR) and 95 % confidence intervals (CI) for poor psychological well-being by exposure to SHS and stratified by age

Age stratification	SHS exposure	Unadjusted		Adjusted^a^	
		OR	95 % CI	OR	95 % CI
<39	no	1	-	1	-
	yes	1.663	1.387–1.994	1.398	1.152–1.696
40–59	no	1	-	1	-
	yes	1.472	1.250–1.733	1.291	1.087–1.535
>60	no	1	-	1	-
	yes	1.439	0.994–2.084	1.393	0.942–2.059

^a^Adjusted for sociodemographic and working condition factors (gender, education, monthly income, balancing income and expenses, marital status, job type, employment type, employment stability, shift working, weekly working time, smoking area status, physical violence, discrimination, and health status)

## Discussion

In this study, we identified associations between SHS exposure and mental health factors by using large-scale representative data from the Korean working population. Results of the present analyses indicate an expected increase in the risk of poor psychological well-being for never smokers who were exposed to SHS among wageworkers. In previous studies, associations between SHS exposure and major depressive disorder, generalized anxiety disorder, attention-deficit, hyperactivity disorder in children and adolescents [10], the association of SHS exposure and such mental stress [8], the association between SHS exposure and depression by checking serum cotinine of smokers and non-smokers. had showed [26]. Although non-smokers didn’t accept equivalent nicotine effect of smokers, if continually exposed to SHS, there will be impact of the effects of low levels nicotine. So we had assumed that SHS exposure of non-smokers will be given the effects of nicotine like smokers, but because of the indirect effects from low levels of nicotine, appeared to health issues such as the direct effects of nicotine does not arise. Examples of the indirect effects, not euphoric state as induced the dopamine pathway of nicotine, discomfort or depression symptoms by SHS exposure [25, 26]. Previous findings didn’t showed the association between SHS exposure and psychological well-being as mental health factor. So, to our knowledge, this is the first study to assess the association between SHS exposure and psychological well-being, there is little to compare with previous studies.

We had studied the association between well-being and sociodemographic factors, included gender, age, education levels, monthly income, balancing income and expenses, marriage status, health status, hypertension, and obesity.

Previous studies drawn from the KCWS showed other results, suggesting that the well-being of all subjects yielded no differences between the genders [36, 38]. But, we found that females tended to have a lower well-being than males. Because we choose non-smoking wageworkers, and perhaps most of non-smokers are women, such factor is likely to have affected our results. Our finding also suggests that subjects who were over 40 years old significantly tended to have a lower psychological well-being. This finding is consistent with a previous study that older subjects tended to have a lower well-being [36] but contradicts the findings that older individuals are happier with their lives than younger individuals [39]. In stratified by age, the unadjusted ORs of poor psychological well-being for all age group were significantly higher for SHS exposure group compared to reference group. Additional adjustment for covariates, did not alter the OR considerably in <39, and 40–59 group. Among the older than 60, however, the OR was not significant. These findings suggest that health problem and dissatisfaction with the future may be contributing factors among old workers.

This study suggests a positive correlation between education levels and psychological well-being and previous findings [36, 38] are consistent with our findings. We found that a positive correlation between monthly income and psychological well-being and subjects who experienced an imbalance between their income and expenses had showed lower psychological well-being than others experienced balance of income and expenses, and previous findings [36, 38] is consistent with our findings. Margelisch et al. [40] assert that married persons are more satisfied with their lives than non-married persons. They used the concept of life satisfaction as a measure of well-being status and life satisfaction was assessed with the 5-item ‘Satisfaction with Life Scale’ with answers on a 7-point scale. There is little research on the association between marriage status and psychological well-being that is rated as WHO five well-being index. Our findings also suggest that not married group had slightly lower scores of psychological well-being than married. Previous studies about assessment of well-being in chronic diseases were known, including of diabetes [31, 41], and chronic heart diseases (e.g. ischemic heart disease, chronic heart failure, atrial arrhythmia, and hypertensive heart disease) [31]. Our findings are consistent with previous studies in that the relationship of SHS exposure and psychological well-being was differentiated according to chronic diseases, including hypertension, and obesity.

Most results of the association between working condition factors and psychological well-being are consistent with previous findings. Previous findings suggest there are no statistically significant differences between shift workers and non-shift workers [36, 38]. But we found that shift workers had statistically lower psychological well-beings than non-shift workers. When smoking areas were not specified, it conferred a lower psychological well-being. To our knowledge, there is little research on the association between assignment of smoking area and psychological well-being and our results may have been associated with SHS exposure. Schütte et al. [37] discussed an association between physical violence (Men: OR = 1.74, 95 % CI: 1.34–2.26, Women: OR = 1.40, 95 % CI: 1.08–1.81) and discrimination (Men: OR = 2.12, 95 % CI: 1.84–2.45, Women: OR = 2.13, 95 % CI: 1.87–2.42) as psychological work factors and represented poor well-being in 34 European countries. In a previous study of Korea, Byun et al. [42] discusses an association between physical violence and depression (OR = 2.86, 95 % CI: 1.54–5.34) and we found the same tendency regarding depression symptoms in other studies [43–46]. Maddox et al. [47] discuss an association between discrimination and distress in women. We also found that the subjects who had experienced physical violence or discrimination at the workplace over the past twelve months had low psychological well-being. The difference with previous research is that we analyzed psychological well-being and physical violence, and discrimination by a cross analysis rather than a logistic analysis, and the results are consistent with previous findings when psychological well-being, depression symptom and distress are placed in the mental health area.

Well-being index has suggested a number of health information. Previous systemic review of the WHO-5 well-being index showed the applicability of the WHO-5 across study fields, includes of endocrinology (e.g. diabetes), depression, stress, psychology, clinical psychometrics, geriatrics, neurology, cardiology, oncology, obstetrics, pain, suicidology, pediatrics, gynecology, ophthalmology, otolaryngology, health economics [48]. There was no previous research revealing any direct association between SHS exposure and psychological well-being and we consider the results of associations of depression, stress and SHS exposure, as well as associations of psychological well-being and depression, stress, or mental disorder for the first time examined the association between SHS exposure and psychological well-being. In addition, we found that SHS exposed workers had lower psychological well-being than no SHS exposed workers. After adjusting for covariates, significant association between SHS exposure and psychological well-being not changed. In stratified by physical health status, among the bad health status group, however, the association was not significant. The effect of bad physical health status may be strong and attenuate the association between SHS exposure and psychological well-being.

There are several limitations to this study. First, although it demonstrates the association between SHS exposure and psychological well-being, causal relationships are hard to be defined. Therefore, it is probable that SHS exposure and change in psychological well-being are in a causal relationship, and it is an issue that needs a confirmation through a cohort study. Second, a reporting bias may also be suspected as this study that relied on self-report measures of both SHS exposure and variable outcome, the bias may lead to an overestimate the associations observed due to common method variance. Third, we did not take into account the “healthy worker effect” during our study, which may operated as workers in poor psychological well-being have left the labor market or changed job. Fourth, this study is not including of quantitative assessment of SHS exposure. Previous studies had used particle meter (PM), salivary cotinine, carbonyl oxide (CO), 4-(methylnitrosamino)-1-(3-pyridyl)-1-butanol (NNAL) as markers of SHS exposure [49]. Therefore, future studies based on our findings need to try utilized with quantitative assessment. Fifth, there are only two items (e.g. hypertension and obesity) related with chronic diseases diagnosed by a doctor in KWCS questionnaire. So we could not consider other possible chronic diseases.

Despite several limitations, our findings in this study provide critical data on associations of psychological well-being and SHS exposure in a nationally representative sample of employees of Republic of Korea. Our findings have important working condition implications for employees. Not only are psychological well-being meaningful as a result by themselves, they also have a capacity to affect overall health and be used as a reference to indicate the current health of an individual [50]. Therefore, we can expect the increase of workers who take care of their overall health as well as mental health by improving the environment of SHS.

## Conclusion

We found that exposure to SHS was associated with poor well-being measured by the WHO-5 well-being index. Our finding indicate the importance of reducing SHS exposure at the workplace for psychological well-being amongst non-smoking wageworkers.
